# Fresh pork microbiota is temporally dynamic and compositionally diverse across meat, contact surfaces, and processing lines in a pork processing facility

**DOI:** 10.1128/aem.00044-25

**Published:** 2025-04-03

**Authors:** A. E. Asmus, T. N. Gaire, K. M. Heimer, K. E. Belk, R. S. Singer, T. J. Johnson, N. R. Noyes

**Affiliations:** 1 Department of Veterinary Population Medicine, University of Minnesota 5635, St. Paul, Minnesota, USA; 2 Hormel Foods Corporation, Austin, Minnesota, USA; 3 Department of Animal Science, Colorado State University 3447, Fort Collins, Colorado, USA; 4 Department of Veterinary and Biomedical Sciences, University of Minnesota 5635, St. Paul, Minnesota, USA; INRS Armand-Frappier Sante Biotechnologie Research Centre, Laval, Quebec, Canada

**Keywords:** fresh pork, food contact surfaces, microbiota, 16S rRNA, food safety

## Abstract

The goal of this study was to analyze the microbial profiles of meat and contact surfaces from two different processing lines in a pork processing plant, using a 16S rRNA gene amplicon sequencing workflow specifically designed to investigate fresh meat and environmental samples throughout a commercial production schedule. Results indicated that the microbiota differed between the meat and contact surface, both across the two processing lines and within each individual processing line. Differences in the microbiota composition were also strongly associated with both the specific processing dates and the time of day during processing. Much of this variation was associated with distinct amplicon sequence variants unique to each processing date and each processing line throughout the day. The abundance of key taxa associated with food safety and spoilage was also temporally dynamic across a production shift and was different between the meat and contact surface. Overall, the results of this study indicate significant differences in the microbial profiles of the meat and contact surfaces between two processing lines within the same plant. These differences are likely influenced by daily variation in processing and sanitation procedures, as well as differences in the design of the processing lines, which appear to affect the microbiotas of both the meat and contact surfaces.

**IMPORTANCE:**

This study provides critical knowledge that can be used as a foundation for tailored processes to improve fresh pork safety and quality, potentially customized to individual processing lines, time points within a shift, and/or production days. Additionally, this study provides a list of potential biological markers associated with food safety and quality that could be used by processors to develop and validate intervention strategies specific to different processing lines.

## INTRODUCTION

Converting living muscle tissue to food is a complex process that involves dynamic pre- and post-mortem biochemical factors associated with the harvest of the animal. Additionally, multiple processing steps during slaughter and boning rely on a network of automation, equipment, and human interaction to provide a safe, wholesome, and highly functional meat product for consumers. Each step within slaughter and boning can influence the microbial ecology of both the equipment used in subsequent processing lines and the meat produced (1–6).

Fresh meat is highly perishable due to its nutrient-rich environment and favorable conditions for the growth of specific spoilage bacteria (7, 8). The core microbiota found in fresh meat is likely derived from two main sources: contamination from the microbiota specific to the animal and from environmental contamination (9). Changes in the fresh pork microbiota associated with this contamination can adversely impact shelf life (3, 10, 11) and food safety (12). Contact surfaces are a common source of spoilage bacteria (10, 11) and can also facilitate the residence of pathogens such as *Salmonella* as components of a biofilm community (12–14). In pork processing plants, contact surface microbiota can vary across processing lines, suggesting that the meat cut and processing activities related to the processing line may influence the microbiota of contact surfaces (15). Moreover, the transfer of microorganisms from contact surfaces to meat has been demonstrated, with the type of surface in contact with the meat influencing this transfer (16–18). Human interaction on the processing line can also affect the fresh pork microbiota, as a high proportion of microorganisms detected on pork carcass samples originated from either the gloves of employees handling the carcass or from common contact surface touchpoints (6, 19, 20).

Current research into fresh pork microbial ecology has typically been limited to environmental sampling of either the pork carcass or processing line. Additionally, the temporal dynamics of the fresh pork microbiota during a processing schedule are not well understood due to the logistical complexity of sampling both the meat and contact surfaces throughout a processing shift and over multiple consecutive processing dates. Therefore, critical gaps exist in our current knowledge of the temporality of the fresh pork microbiota and how the microbial ecology of the contact surface may influence the meat over the course of a production schedule. The objective of this study was to fill these research gaps by characterizing the microbial ecology of both the contact surface and meat from two distinct processing lines in a single plant, across multiple production dates and throughout a production shift. To facilitate this objective, the study developed a method enabling a broad analysis of meat trim surfaces using a sample size 12 to 200 times larger than what has been traditionally used in 16S rRNA amplicon sequencing workflows, while still accommodating the sampling constraints of a commercial production schedule.

## RESULTS

### Sequencing results

The mean 16S rRNA amplicon copy number was 854 copies/μL for all samples (range: 1–167,444, median = 296). For meat samples, the 16S rRNA copy number was 1,449 copies/μL (range: 1–167,444) and for contact surface samples 260 copies/μL (range: 3–2,273). The mean copy number for the negative controls was two copies/μL (range: 0–3) and 31,466 and 35,605 copies/μL for the two positive controls. Seven percent of meat samples and 40% of contact surface samples had a copy number of less than 100 copies/μL, indicating that these can be considered low biomass samples.

A total of 78.6 M paired-end 16S rRNA gene reads were generated across all 394 meat samples, contact surface samples, and negative and positive controls (Fig. S1). The average number of raw reads was 209,966 reads per meat sample (range 1,416–377,400), 191,696 reads per contact surface sample (range 11,543–318,464), 171,411 reads per negative control (range 63,797–261,589), and 95,464 and 112,880 reads for the two positive controls. A total of 33.7 M reads remained after quality control filtering and merging forward and reverse reads in the DADA2 package. A majority of the reads were removed by the filterAndTrim function in DADA2*,* with the highest proportion of reads removed in samples that contained a high percentage of adapter content in the sequence. The percentage of reads that remained after using filterAndTrim ranged from 44.6% to 82.5% per sample.

The two positive controls generated 208,344 total reads and 140,316 remained after quality control analysis in DADA2. Seven genus-level taxa were assigned to the sequences in the positive control data (Fig. S2). *Staphylococcus* was not recovered in either mock community control, likely due to the low overall relative abundance in the mock control (0.0007%). Additionally, *Pseudomonas* was overrepresented at 40% of the mock positive controls (theoretical abundance = 2.8%), while *Listeria* was underrepresented at 50% of the mock positive controls (theoretical abundance = 95.9%). The overrepresentation of *Pseudomonas* may be an indication of potential bias that may exist in the methodology, possibly due to extraction biases and/or the 16S rRNA gene forward primer used for amplification; copy number variation of the 16S rRNA gene within individual genomes can also skew relative abundance estimations.

Within the package decontam, the Combined method was used to identify 2,438 amplicon sequence variants (ASVs) as potential contaminants, leaving 127,437 ASVs comprising 31.4 M reads for further analysis (Fig. S1). The composition of contaminants identified and removed by the Combined method is described in Fig. S3. No significant differences were observed between the average number of reads generated for meat or contact surface sample types (Fig. S1) after the removal of ASV contaminants. At the Domain level, ASVs were classified as Bacteria (92.6%), Archaea (0.1%), Eukaryota (0.01%), and “NA” (7.3%). The percentage of reads classified against the SILVA 138.1 database decreased as the taxonomic level decreased, resulting in a 62% read classification rate at the genus level, but only 9.8% at the species level (Table S1). Classification rates for ASVs were even lower, with 23.2% of all ASVs identified at the genus level and only 1.3% identified at the species level (Table S1). Based on these results, microbiota analysis was conducted at the genus level.

### Microbiota variation is associated with processing date, processing line, and sample type

The variation in beta diversity at the ASV level attributed to each study variable was statistically significant for all individual factors (Table 1). In terms of effect size, processing date (*R*^2^ = 0.08, *P* = 0.001), processing line (*R*^2^ = 0.07, *P* = 0.001), sample type (*R*^2^ = 0.06, *P* = 0.001), and shift time (*R*^2^ = 0.02, *P* = 0.001) were associated with 23% of the total explained variation in the permutational multivariate analysis of variance (PERMANOVA) model. Visualization of beta diversity by non-metric multidimensional scaling (NMDS) ordination revealed significant clustering by processing line and sample type, suggesting that the overall microbiota is dissimilar between both the Bootjack Trim (BJ) and Boston Butt Trim (BBT) processing lines and between the meat and contact surface sample types (Fig. S4A and B). Analysis of beta dispersion revealed significant differences (*P* = 0.001) by sample type (Fig. S4C). The BBT meat samples had the farthest average distance to the median (0.50), while the BJ contact surface samples had the smallest average distance to the median (0.46). Meat samples from both the BJ and BBT (0.47 and 0.50, respectively) had higher average distances to the mean than contact surface samples from the BJ and BBT (0.46 and 0.48, respectively).

**TABLE 1:** Variance in beta diversity associated with key factors across all samples and at each level of processing line and sample type (PERMANOVA)^*a*^

Factor	*N*	*R* ^2^	*P*-value
All samples			
Processing date	16	0.08	0.001
Processing line (BJ or BBT)	2	0.07	0.001
Sample type (meat or contact surface)	2	0.06	0.001
Shift time (beg or end)	2	0.02	0.001
Total *R*^2^		0.23	
Bootjack Trim—meat			
Processing date	16	0.24	0.001
Shift time (beg or end)	2	0.03	0.001
Bootjack Trim—contact surface			
Processing date	16	0.34	0.001
Shift time (beg or end)	2	0.06	0.001
Boston Butt Trim—meat			
Processing date	16	0.25	0.001
Shift time (beg or end)	2	0.05	0.001
Boston Butt Trim—contact surface			
Processing date	16	0.23	0.001
Shift time (beg or end)	2	0.07	0.001

^*a*^ Fixed effects and number of levels of each factor are designated in the Factor and N columns, respectively. The estimate of beta diversity explained by each factor is reported in the *R*^2^ column. The statistical significance of each *R*^2^ estimate is reported in the *P*-value column.

### Meat and contact surface samples contain significantly different microbiota

**Fig 1:**
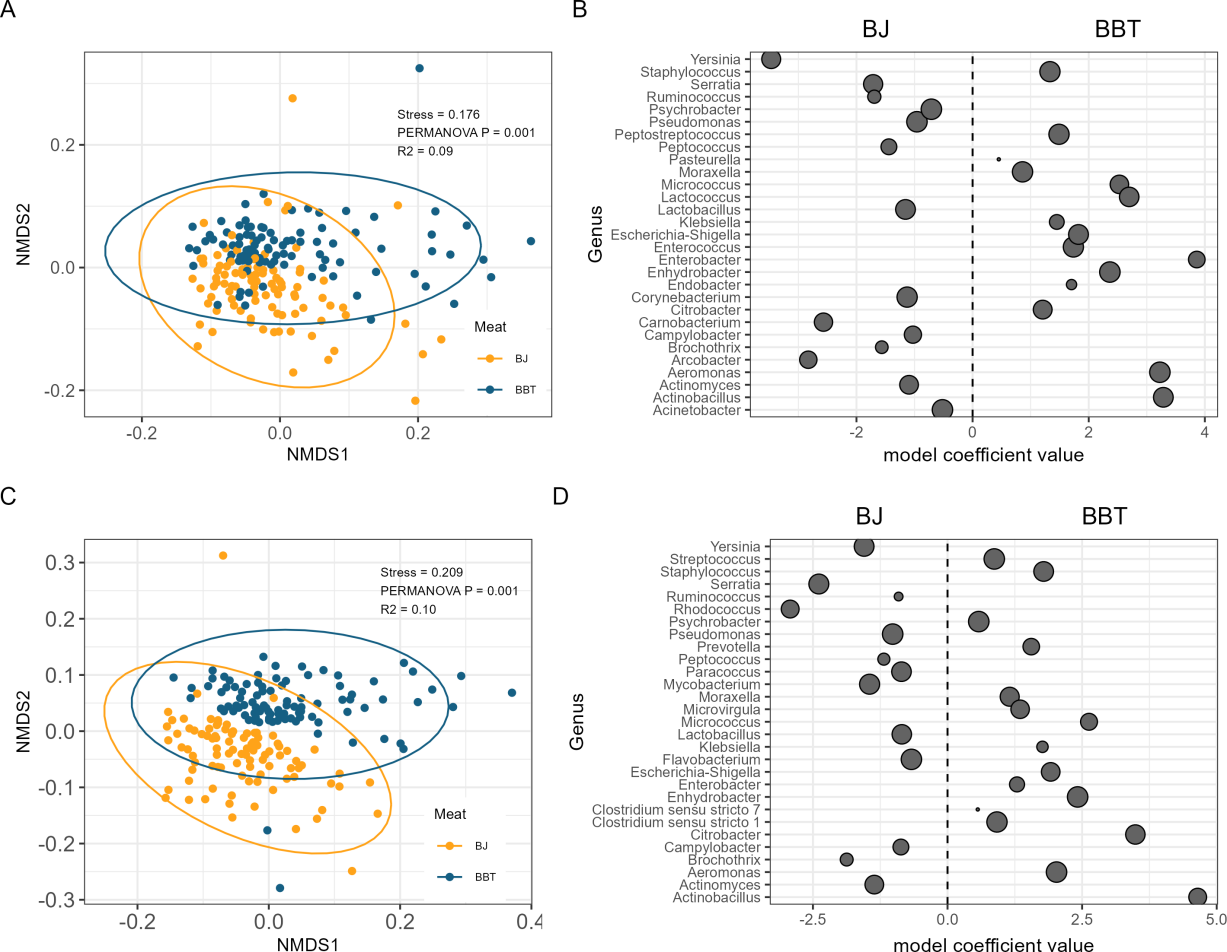
Compositional description of the microbiota by processing line. (A) Non-metric multidimensional scaling ordination plot of Bray-Curtis dissimilarities of meat samples from Bootjack Trim (yellow) and Boston Butt Trim (blue). (B) Differences in abundance of select microbial genera between meat samples from the Bootjack Trim (negative coefficient) and Boston Butt Trim (positive coefficient) that are contributors to food safety or shelf life. (C) Non-metric multidimensional scaling ordination plot of Bray-Curtis dissimilarities of contact surface from Bootjack Trim (yellow) and Boston Butt Trim (blue). (D) Differences in abundance of select microbial genera between contact surface samples from the Bootjack Trim (negative coefficient) and Boston Butt Trim (positive coefficient) that are contributors to food safety or shelf life. Each dot represents a microbial genus that is significantly different between the two processing lines, with the size of the dot representing the overall prevalence of the genus.

**Fig 2:**
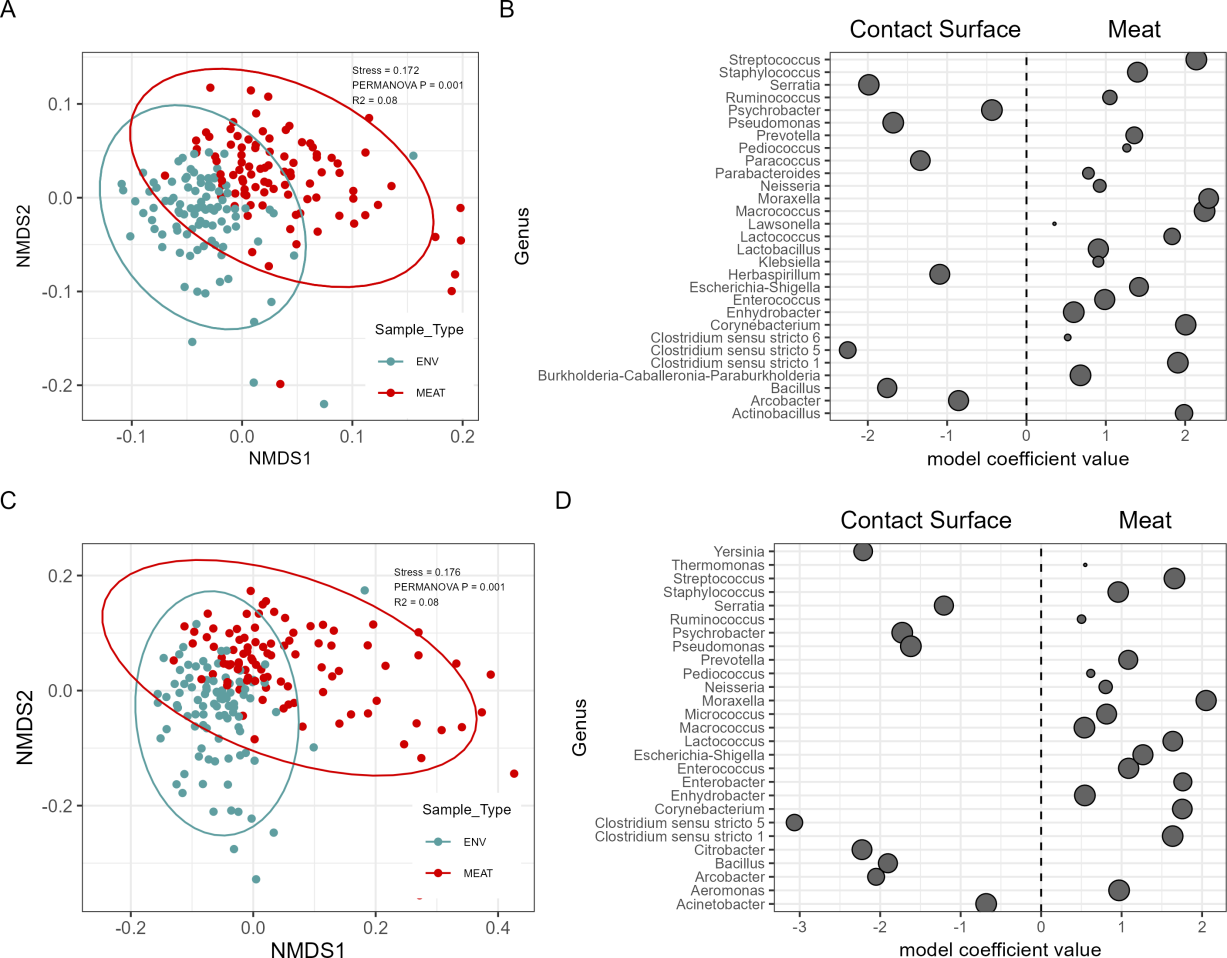
Compositional description of the microbiota by sample type. (A) Non-metric multidimensional scaling ordination plot of Bray-Curtis dissimilarities of Bootjack Trim samples from the contact surface (gray) and meat (red). (B) Differences in abundance of select microbial genera between Bootjack Trim from the contact surface (negative coefficient) and meat (positive coefficient) that are contributors to food safety or shelf life. (C) Non-metric multidimensional scaling ordination plot of Bray-Curtis dissimilarities of Boston Butt Trim samples from the contact surface (gray) and meat (red). (D) Differences in abundance of select microbial genera between Boston Butt Trim from the contact surface (negative coefficient) and meat (positive coefficient) that are contributors to food safety or shelf life. Each dot represents a microbial genus that is significantly different between the two sample types, with the size of the dot representing the overall prevalence of the genus.

Visualization of beta diversity by NMDS ordination at the ASV level revealed significant clustering by processing line in both the meat (*R*^2^ = 0.09, *P* = 0.001) and contact surface (*R*^2^ = 0.10, *P* = 0.001), suggesting dissimilarity between the microbiota of the two processing lines (Fig. 1A and C). Additionally, significant clustering between the meat and contact surfaces was observed within the BJ (*R*^2^ = 0.08, *P* = 0.001) and the BBT (*R*^2^ = 0.08, *P* = 0.001) processing lines (Fig. 2A and C). This would suggest that in addition to the dissimilarity between the two processing lines, the microbiota of the meat and contact surfaces within each processing line are also dissimilar.

Differential abundance of genera between groups of samples was determined using normalized ASV counts aggregated at the genus level. The abundances of 207 genera were found to be significantly different between the meat samples from each processing line, with 57 genera more abundant in the BBT meat and 150 genera more abundant in the BJ meat (Fig. 1B; Data set S1, “DiffAb_Meat.csv”). The abundances of 193 genera were found to be significantly different between the contact surface samples from each processing line, with 56 genera more abundant in the BBT contact surface and 137 genera more abundant in the BJ contact surface samples (Fig. 1D; Data set S2, “DiffAb_Env.csv”). *Yersinia, Serratia, Pseudomonas, Lactobacillus,* and *Campylobacter* had higher relative abundance in the BJ compared to the BBT samples, while *Staphylococcus*, *Moraxella, Micrococcus, Lactococcus, Enterococcus, Escherichia-Shigella,* and *Aeromonas* all had higher relative abundance in the BBT vs BJ.

Within the BJ processing line, the abundances of 183 genera were found to be significantly different between the meat and contact surface, with 124 more abundant in the meat and 59 more abundant in the contact surface at the qval < 0.05 level (Fig. 2B; Data set S3, “DiffAb_BJ.csv”). *Streptococcus, Staphylococcus, Moraxella, Lactobacillus, Lactococcus, Escherichia-Shigella, Enterococcus,* and *Clostridium sensu stricto 1* (CSS1) were all of higher abundance in the BJ meat versus contact surface, while *Serratia, Pseudomonas, Paracoccus,* and *Clostridium sensu stricto 5* were more abundant in the BJ contact surface versus the meat.

The abundances of 144 genera were found to be significantly different between the meat and contact surface samples from the BBT processing line, with 85 genera more abundant in the BBT meat and 59 genera more abundant in the BBT contact surface at the qval < 0.05 level (Fig. 2D; Data set S4, “DiffAb_BBT.csv”). *Streptococcus, Staphylococcus, Moraxella, Micrococcus, Escherichia-Shigella, Enterococcus, Aeromonas,* and CSS1 had higher relative abundance in the BBT meat compared to the BBT contact surface samples, while *Yersinia, Serratia, Pseudomonas, Clostridium sensu stricto 5, Citrobacter, Bacillus,* and *Acinetobacter* all had higher relative abundance in the BBT contact surface samples.

**Fig 3:**
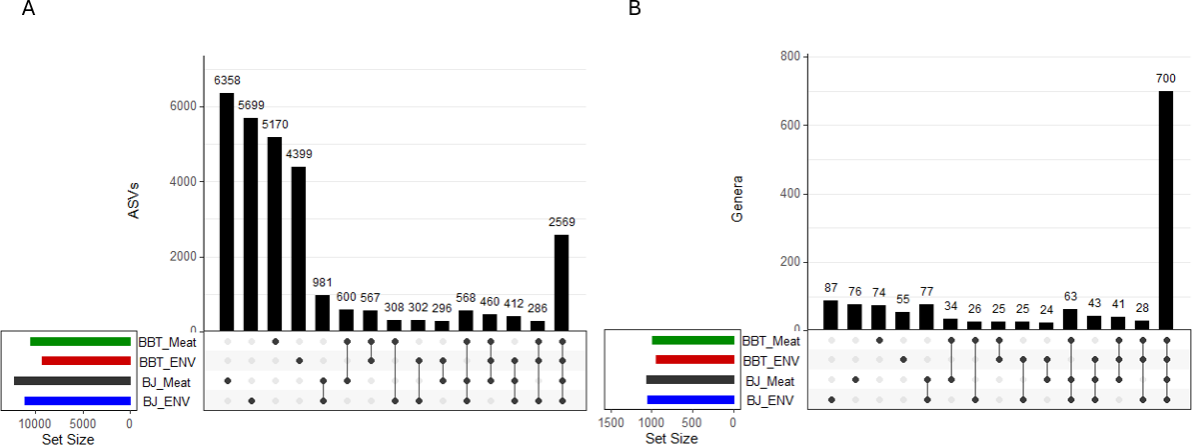
UpSetR plots showing the intersection of common (A) ASVs and (B) genera across processing lines (BJ and BBT) and sample type (meat and contact surface). The total number of intersecting ASVs (A) and genera (B) is represented on the *y*-axis. Each level of processing line and sample type is denoted on the *x*-axis, with dots representing the combination of each factor included in the total number of intersecting ASVs (A) or genera (B) reflected in each bar. The total number of ASVs (A) and genera (B) for each level of processing line and sample type is reflected in the Set Size bar chart. ASVs were agglomerated to individual genera using tax_glom in the phyloseq package.

To give further context to the beta diversity measurements, UpSet plots were used to visualize the intersections of individual ASVs and ASVs aggregated by genus across the processing lines and sample types (Fig. 3). Intersecting ASVs (i.e., ASVs identified in multiple sample types) accounted for 21%–27% of the total ASVs across each combination of processing line and sample type. In contrast, non-intersecting ASVs (i.e., ASVs that were unique to a given sample type) ranged from 47.3% to 51.2% of total ASVs. Within processing lines, the percentage of ASVs shared between meat and contact surface samples was 8.0%–8.8% in the BJ Line and 5.4%–6.1% in the BBT Line. The amount of intersection generally increased when performing this analysis at the genus level. For example, 700 genera intersected across all processing lines and sample types, representing 66.2%–74.3% of the total genera for each combination of processing line and sample type (as compared to 21%–27% for ASVs). Conversely, the proportion of non-intersecting genera was lower than that of non-intersecting ASVs, ranging from 5.8% to 8.3%. Genera unique to the meat or contact surface in the BJ processing line ranged from 7.2% to 7.3%, while in the BBT processing line they represented 2.5%–2.7%.

### Meat and contact surface microbiota change significantly throughout a single processing shift

ASV-level Bray-Curtis dissimilarities were calculated by shift time for each combination of processing line and sample type (Table 1). Shift time was significantly associated with beta diversity variation in samples from the BJ meat (*R*^2^ = 0.03, *P* = 0.001), BJ contact surface (*R*^2^ = 0.06, *P* = 0.001), BBT meat (*R*^2^ = 0.05, *P* = 0.001), and BBT contact surface (*R*^2^ = 0.07, *P* = 0.001). This suggests that the microbiota composition of the meat and contact surfaces changes during the shift.

Intersections of ASVs classified at the genus level by processing line across the production shift were visualized using UpSet plots (Fig. S5). In the BJ processing line, 2,012 ASVs were shared between the meat and contact surfaces across both shift times, representing 25.2%–29.6% of the total ASVs at each time point. ASVs unique to each shift time in either meat or contact surface accounted for 42.9%–46.0% of the total ASVs analyzed. A similar trend was observed in the BBT processing line, where 23.4%–29.2% of ASVs were shared between BBT meat and contact surfaces across the production shift, while unique ASVs at each shift time point represented 44.0%–48.4% of the total ASVs in either the meat or contact surface.

### Key taxa associated with food safety and quality are differentially abundant between processing lines and across the processing shift

**Fig 4:**
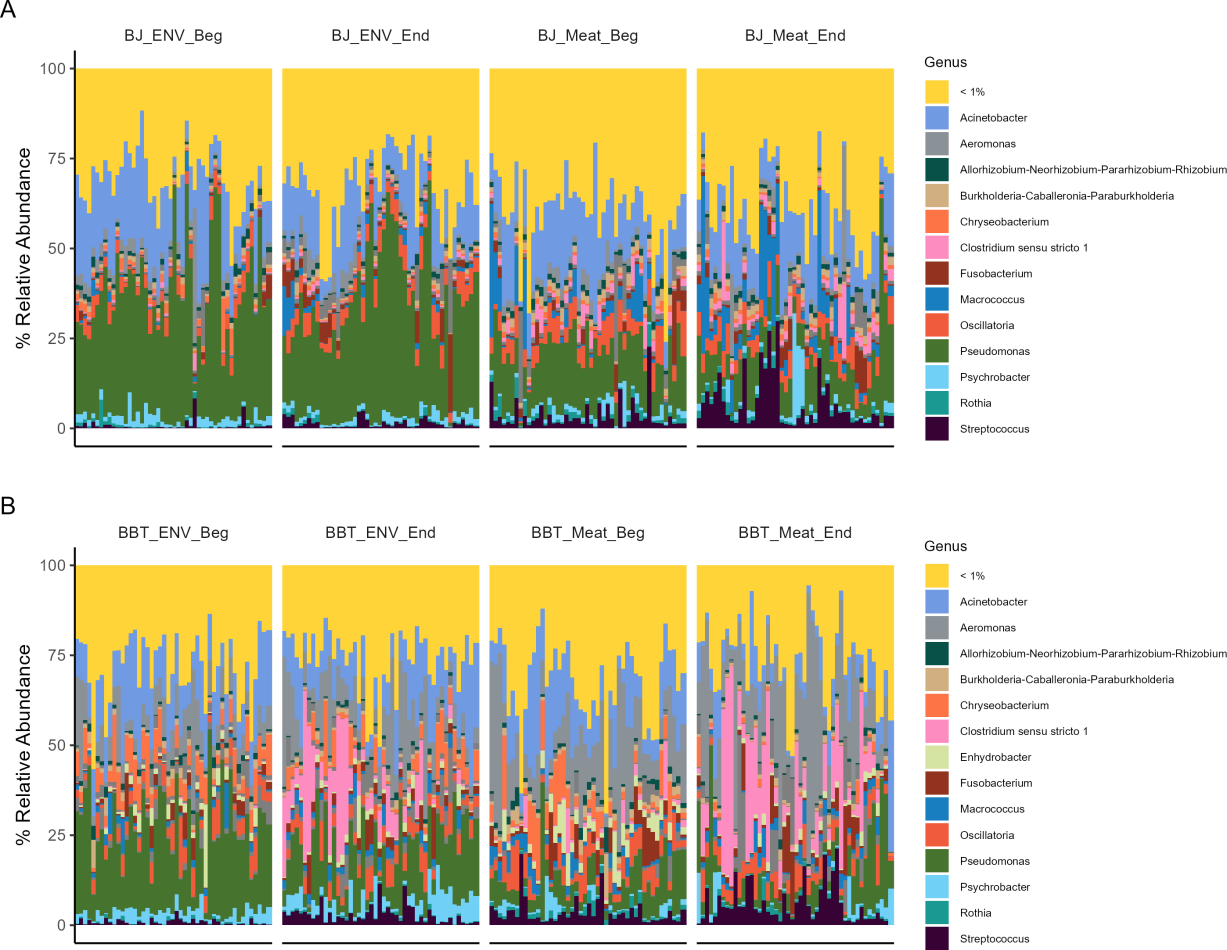
Taxonomic abundance across the production shift at the genus level. Each individual bar denotes an individual sample. (A) Taxonomic relative abundance of individual Bootjack Trim contact surface (ENV) and meat samples. (B) Taxonomic relative abundance of individual Boston Butt Trim contact surface (ENV) and meat samples. Samples with less than 1% median relative abundance are cumulatively reported as “<1%.”

**Fig 5:**
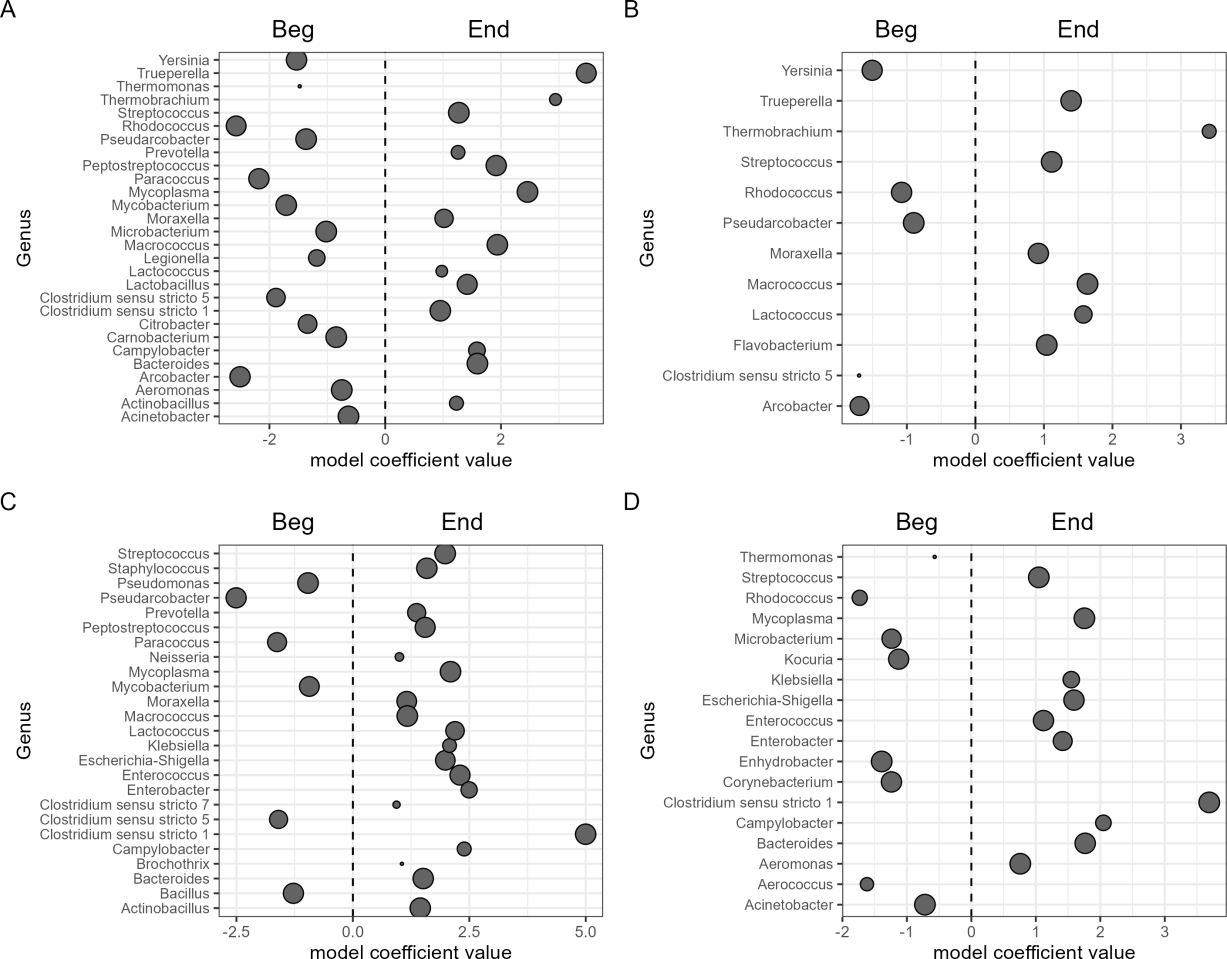
Differences in abundance of select genera that are contributors to food safety and shelf life across the production shift. (A) Differences of microbial genera in Bootjack Trim contact surface between the beginning (negative coefficient) and end (positive coefficient) of the production shift. (B) Differences of microbial genera in Bootjack Trim meat between the beginning (negative coefficient) and end (positive coefficient) of the production shift. (C) Differences of microbial genera in Boston Butt Trim contact surface between the beginning (negative coefficient) and end (positive coefficient) of the production shift. (D) Differences of microbial genera in Boston Butt Trim meat between the beginning (negative coefficient) and end (positive coefficient) of the production shift. Each dot represents a microbial genus that is significantly different between the two processing lines, with the size of the dot representing the overall prevalence of the genus.

Notable differences in the abundance of individual genera were observed across the production shift in both the BJ and BBT processing lines when ASVs were grouped by genus (Fig. 4). The abundance of taxa such as *Streptococcus, Flavobacterium, Moraxella*, and *Lactococcus* was higher at the end of the shift compared to the beginning in both the BJ meat and contact surface samples (Fig. 5A and B). However, *Streptococcus, Klebsiella, Escherichia-Shigella, Enterobacter, Campylobacter,* and *Bacteroides* had significantly higher abundance at the end of the shift compared to the beginning, in both the contact surface and meat samples from the BBT processing line (Fig. 5C and D).

Differences in the relative abundance of the genus CSS1 were also observed from beginning to end of shift on both processing lines and in both sample types (Fig. 4A and B). In the BJ contact surface samples, CSS1 abundance was significantly higher at the end of the shift as compared to the beginning of the shift (model coefficient = 0.95, qval = 0.001, Fig. 5A). However, the abundance of CSS1 was not significantly different between the beginning and end of the shift in the BJ meat (model coefficient = 0.78, qval = 0.30). Within the BBT processing line, a large and significant increase in the abundance of CSS1 was observed between the beginning and end of the shift in the contact surface samples (model coefficient = 5.0, qval = <0.0001, Fig. 5C) and in the meat samples (model coefficient = 3.7, qval = <0.0001, Fig. 5D). Additionally, CSS1 had the highest model coefficient value among all genera analyzed from the beginning to the end of the shift on both the BBT meat and contact surfaces, indicating that CSS1 experienced the greatest change in abundance throughout the shift.

While a majority of CSS1 ASVs were unable to be classified at the species level, the crude prevalence of ASVs classified as *Clostridium butyricum* was higher in the BJ meat vs BBT meat (89% vs 66%) and in the BJ contact surface vs BBT contact surface (56% vs 30%). However, the crude prevalence of ASVs identified as *Clostridium perfringens* was higher in the BBT meat vs BJ meat (52% vs 7%) and in the BBT vs BJ contact surface (41% vs 2%). Crude prevalence of *C. perfringens* also increased from the beginning to the end of the shift in the BBT meat (11% to 92%) and in the BBT contact surface (2% to 81%).

**Fig 6:**
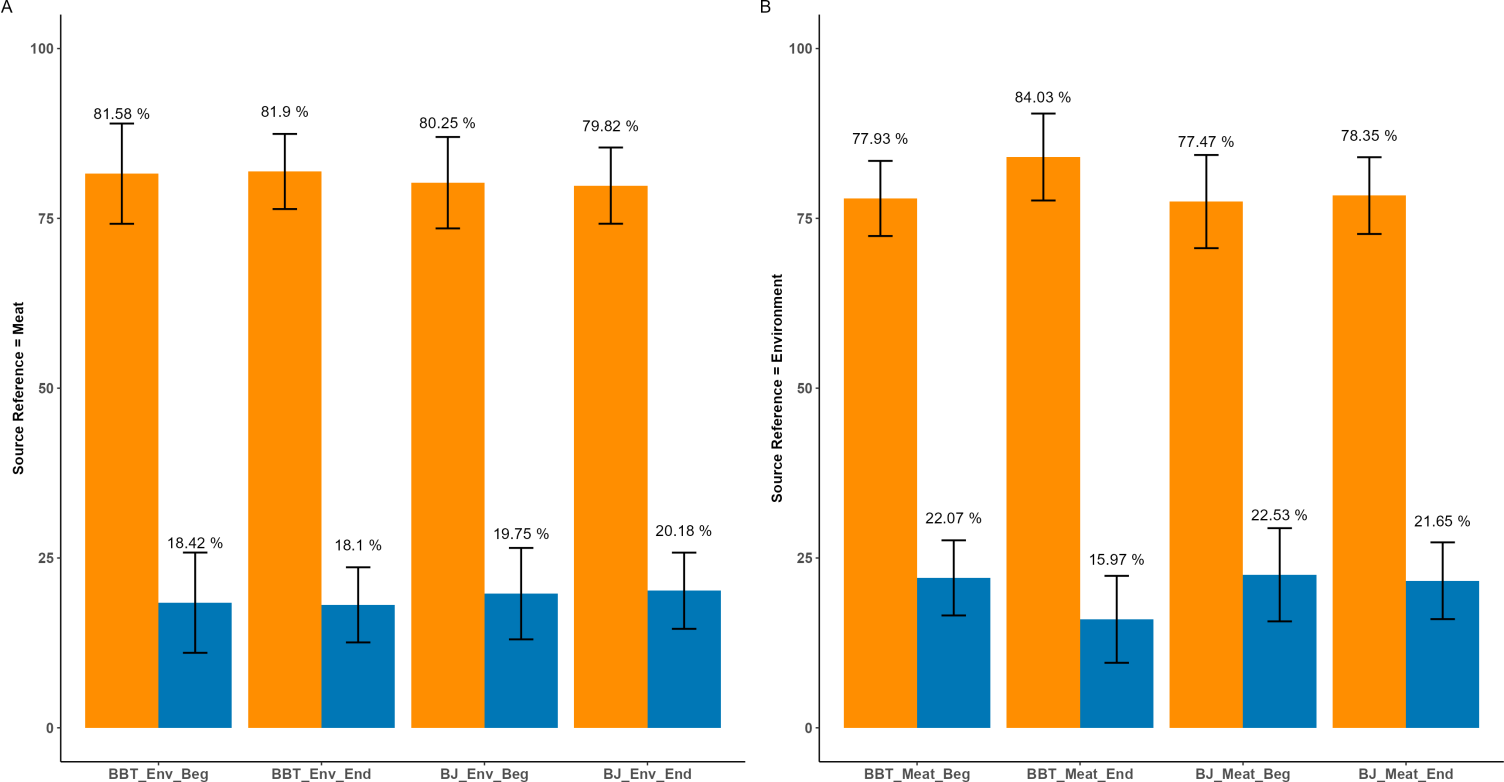
Potential sources of bacteria in meat and contact surface samples (SourceTracker). (A) Proportion of potential meat source communities (orange) and unknown source communities (blue) on contact surface samples by processing line (BJ and BBT) and across a production shift. (B) Proportion of potential contact surface source communities (orange) and unknown source communities (blue) on meat samples by processing line (BJ and BBT) and across a production shift.

To identify potential sources of the microbes identified in the meat and contact surface samples in each processing line, SourceTracker analysis of individual ASVs was performed (Fig. 6). The results were remarkably consistent across both processing lines and the production shift. Meat contributed 79.8%–81.6% of the microbial population in the contact surface samples across both processing lines and the production shift, with the remainder coming from an unknown source. Similarly, the contact surface accounted for 77.4%–84.0% of the microbial population in the meat samples across both processing lines and the production shift, with the rest attributed to an unknown source. These findings suggest that, despite differences in microbial diversity and the abundance of individual taxa throughout the shift, the meat and contact surfaces contribute significantly to each other’s microbial populations in a consistent manner across processing lines and shift times. The relatively large fraction of bacteria from an unknown source further suggests that microbial sources beyond what was measured in this study may play a key role in establishing the microbes found in meat and contact surface samples.

### Unique ASVs contribute to microbiota variability across individual processing dates

ASV-level Bray-Curtis dissimilarities were calculated by processing date for each combination of processing line and sample type (Table 1). The processing date was significantly associated with a relatively large proportion of total beta diversity variation in the BJ meat (*R*^2^ = 0.24, *P* = 0.001), BJ contact surface (*R*^2^ = 0.34, *P* = 0.001), BBT meat (*R*^2^ = 0.25, *P* = 0.001), and BBT contact surface (*R*^2^ = 0.23, *P* = 0.001). NMDS ordination of the Bray-Curtis dissimilarities showed that the centroids of samples from each processing date were non-overlapping, despite large sample to sample variation (i.e., high beta dispersion) (Fig. S4).

**Fig 7:**
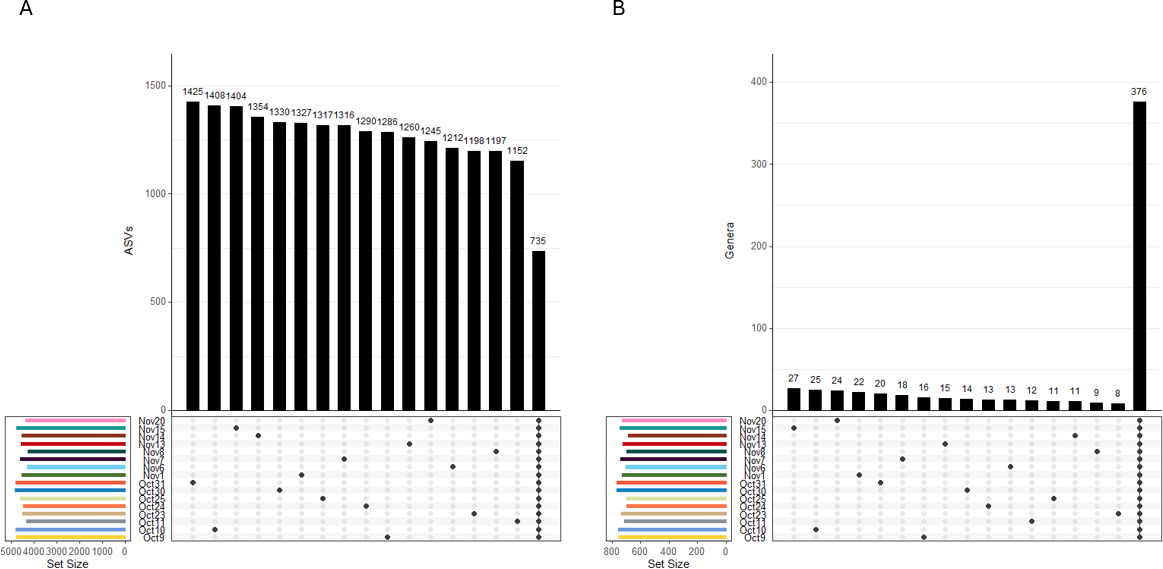
UpSetR plots showing the intersection of individual (A) ASVs and (B) genera across processing dates. ASVs were agglomerated to individual genera using tax_glom in the phyloseq package. The total number of intersecting ASVs (A) and genera (B) is represented on the *y*-axis. Each individual processing date is denoted on the *x*-axis, with dots representing either each individual date or all dates combined included in the total number of intersecting ASVs (A) or genera (B) reflected in each bar. The total number of ASVs (A) and genera (B) for each processing date is reflected in the Set Size bar chart. Each processing date includes all samples from both processing lines (BJ and BBT) and sample types (meat and contact surface).

To provide further context to the beta diversity measurements associated with processing date, intersections of individual ASVs identified at the genus level and ASVs grouped together by genus on individual processing dates were visualized using UpSet plots (Fig. 7). The total number of individual ASVs and genera identified on each production date ranged from 4,265 to 4,820 ASVs and 686–765 genera. ASVs that were unique to individual processing dates (i.e., non-intersecting ASVs) encompassed 27%–30% of total ASVs observed. ASVs found across all processing dates represented 15%–17% of total ASVs. In contrast, when ASVs were grouped by genus, most genera were shared across all processing dates (49%–54%), while only 1%–4% were unique to individual processing dates, i.e., non-intersecting. ASVs that were common across all 16 processing dates tended to have the highest abundance out of all ASVs. For example, 548 ASVs were classified as *Pseudomonas,* and of the 30 most abundant *Pseudomonas* ASVs, 28 were identified across all 16 production dates. A similar trend was observed for other common genera such as *Aeromonas* and CSS1. Taken together, these results suggest that while daily variation at the genus level may be less common, variation in individual ASVs within these genera is common, and often associated with individual processing dates.

## DISCUSSION

### Early establishment and differentiation of meat-associated microbiota

Our study using 16S rRNA gene amplicon sequencing revealed that both meat and contact surfaces from different processing lines harbor highly diverse yet distinct microbial communities (Fig. 1 and 2), suggesting that differences in microbiota may begin early post-slaughter. Supporting this, Braley et al. (21) reported site-specific microbiota on pork carcasses, noting that enterobacteria were more prevalent on the shoulder than on the ham (21). However, the authors found similar overall microbiota composition between these carcass sites, emphasizing the need to assess microbiota differentiation in later processing stages.

Our results indicate that microbial populations become increasingly differentiated during further processing of primal cuts into subprimal cuts and trim meat, in particular across the BJ and BBT processing lines. A recent study by Shedleur-Bourguignon et al. (15) found significant differences in contact surface microbiota alpha and beta diversity between the loin, picnic shoulder, belly, and ham processing lines in the same processing plant (15). However, these authors did not report on paired meat samples, and it was not known whether the distinct microbiota found across the contact surfaces of different lines would also be reflected in the subprimal meat cuts on those same lines. Our inclusion of paired trim meat and contact surface samples allowed us to identify cross-line differentiation of microbial populations in both contact surfaces and meat samples. This observation may help processors develop stage-specific interventions in processing lines, potentially improving food safety and quality outcomes by targeting bacteria associated with spoilage and contamination.

### Factors influencing microbial differentiation along the processing chain

Several factors likely contribute to the differentiation of microbial populations, including distinct bacterial communities on the skin of live animals (4, 20, 22), variability in the post-slaughter process (1, 5, 22, 23), and differences in environmental and handling conditions across processing lines (6, 24, 25). Variability between the BJ and BBT lines may stem from differences in the degree of meat handling, exposure to workers, and interaction with transfer belts and surfaces. For example, meat on the BBT line had increased contact with workers and additional processing equipment, which may have promoted higher microbiota variability compared to the BJ line (Fig. 1 and 4), which had fewer handling steps and less exposure to processing equipment. The cumulative exposure to these line-specific processing environments highlights the importance of tailored interventions for each line. To further understand upstream factors contributing to differentiated microbial populations in trim products, future studies should include sampling at multiple stages within the slaughter and boning processes.

### Temporal variability and potential implications for product shelf life

Of all the factors measured in our study (i.e., sample type, processing line, shift time, and sampling date), processing date was associated with the largest amount of microbiota variability across all samples (Table 1). Shelf life variability remains a persistent challenge, often caused by specific spoilage bacteria that intermittently contaminate raw ingredients during harvest, processing, and packaging, leading to faster spoilage and increased food waste (3, 10, 26, 27). Hultman et al. (28) found that the microbial composition varied between equipment, meat, and cooked sausages (28). However, common operational taxonomic units were detected throughout the raw to cooked process and were also detected at the end of the cooked sausage shelf life, suggesting that potential spoilage bacteria can be introduced by the raw trim meat and/or raw processing environment (28). A better understanding of the variability and contribution of raw ingredient microbial profiles (such as trim) and the processing environment could improve product quality consistency and reduce shelf life failures.

Sources of variation exist throughout the entire post-slaughter process; any one of which could contribute to differences in the carcass surface microbiota and the plant environmental microbiota, ultimately leading to the date-associated variability we observed in the meat and contact surface samples (2, 4, 5, 20, 29). Interventions that target this daily variability upstream from the primary processing line should be considered as a potential priority for reducing variability in spoilage organisms in raw meat. Examples include interventions that target the carcass surface microbiota as it transitions from evisceration to the carcass cooler (30, 31) and also targeted sanitation of the cutting equipment throughout the evisceration process to minimize the risk of cross-contamination between carcasses (32–34).

It should be noted that this study was conducted over a 6-week period at one pork processing plant. Given the processing differences that exist across plants, it is unclear if similar spatial and temporal trends would exist in other plants or across different seasons throughout the year. Additionally, this study was conducted only during the first shift of production, and the study facility ran an additional shift that began immediately after the end of the first shift. At the end of the second shift, the facility undertook a daily, complete sanitation that was completed prior to the start of the first shift on the following day. Thus, this study provides insight into the temporal dynamics across the first half of each production date, but does not provide an assessment of what may be occurring in the second half of each production date.

### Intra-shift temporal dynamics as biomarkers for targeted interventions

The temporal dynamics observed at the ASV level suggest that certain bacterial genera, including CSS1, increase in abundance over the course of a shift. This trend, particularly prominent on the BBT line, supports the hypothesis that meat serves as a source for genera such as CSS1, *Streptococcus*, *Escherichia coli-Shigella*, *Klebsiella*, and *Enterobacter*. The BBT line’s design—with more handling and exposure points—likely contributes to the higher abundance of these bacteria compared to the BJ line. Specifically, the BBT processing line had more handling, more individual cuts made from the primal cut, more exposure to workers, more exposure to multiple transfer belts, and a common trim accumulation location. All of these factors could have contributed to the different intra-shift dynamics observed in the BBT versus the BJ samples. Campos Calero et al. also found that the bacterial communities within a pork processing plant were significantly different by location and also influenced by hygienic conditions in the processing plant (2); and previous work has shown that workers’ gloves can contribute significantly to the microbiota of fresh meat (19, 20). Our ASV-level results would suggest that a targeted intervention such as mid-shift sanitation or sanitary design improvements to each processing line to either reduce or limit the increase of bacteria throughout the shift may improve the overall food safety and quality risk profile of these trim materials, especially on the BBT line. Future studies could assess whether such strategies reduce microbial loads and improve food safety profiles without imposing additional costs or downtime.

### Insights from sub-genus-level microbiota analysis in meat processing

16S rRNA gene amplicon sequencing enabled us to detect and characterize key bacterial taxa that impact food safety and spoilage. Unlike traditional culture-based methods, which may miss lower-abundance taxa or fail to resolve taxonomic details, 16S rRNA gene amplicon sequencing can offer a comprehensive analysis of the fresh pork microbiota. This technique allows for semi-quantitative insights into individual taxa presence and abundance, providing processors with valuable information on bacterial dynamics that can support targeted interventions. Additionally, our study utilized an efficient, novel process and application of the MicroTally spun polymer cloth mitt to gain further understanding of the microbiota on trim meat surfaces. The method described in this study allowed for the analysis of approximately 1,600–2,000 g of meat per sample, which is 12 to 200 times greater than traditional meat sample preparation methods used in 16S rRNA gene amplicon sequencing workflows.

Our results suggest that variability within the microbiota exists beyond the genus level, with important implications for food processing. Species- and strain-level differences within genera such as *Clostridia* and *Pseudomonas* impact spoilage mechanisms (35–37), thermal resistance (38), and biofilm formation on contact surfaces (39, 40), underscoring the need for taxonomically resolved microbiota analysis. Such nuanced changes in the microbiota may not be fully recognized by “broad spectrum” culture-based methods such as aerobic plate count, *Enterobacteriaceae* count plates, or qPCR for targeted bacteria. A further understanding of these species- and strain-level dynamics will be crucial for designing effective interventions to improve the quality and safety of fresh pork.

### Conclusion

Taken in total, this study presents evidence that the microbial ecology of the meat and contact surfaces is dissimilar between two separate processing lines and is also dissimilar within the processing line. Additionally, the temporal dynamics of key taxa associated with food safety and product quality provide insight into potential biological markers that could be used to assess strategies for targeted intervention on each processing line. While it is impossible for meat processors to control all sources of microbial variation during slaughter and further processing, this study underscores the importance of reducing points of variation that contribute to microbiota differences. Effectively managing these factors, along with implementing targeted interventions to limit microbial variation, could improve both food safety and the shelf life of fresh pork products.

## MATERIALS AND METHODS

### Study design and sample collection

Meat and contact surface swab samples were collected from two individual fresh pork processing lines at a large-scale pork processing facility that harvested approximately 10,000 market hogs per shift. This production facility conducted three 8 hour production shifts throughout each production date. The first two 8 hour shifts were production processing shifts, and a full sanitation occurred during the last 8 hour shift. A 25 ppm chlorine rinse of the carcass was incorporated after carcass evisceration and prior to the carcass entering the chiller, where the carcass was chilled to 4.5°C in approximately 24 hours according to industry standards. No other antimicrobials were used on either the carcass or processing lines during normal production.

Samples collected for this study were obtained from the belly (item #408 [41]) and shoulder (item #403 [41]) primal cut processing lines, with the specific meat samples being collected from Bootjack Trim and Boston Butt Trim cuts. BJ was removed as a water jet knife made a perpendicular cut on the posterior end of the belly to meet item #408 finished meat specifications (41) and sized for target dimensions required for further processing. After the belly was cut, the BJ was immediately transferred via a conveyor to a corresponding 900 kg stainless steel vat. BBT was removed from the Boston butt shoulder (item #406 [41]) as it was trimmed at individual workstations via a Whizard knife on the peripheral and anterior ends of the shoulder to meet item #406 finished meat specifications (41). After the BBT was removed from the Boston butt shoulder, it dropped to a conveyor belt that transferred it to a stainless steel gathering chute through which the BBT fell directly into a corresponding 900 kg stainless steel vat.

Sampling of both the contact surface and meat samples occurred 3 days per week (Monday, Tuesday, and Wednesday) for 5 consecutive weeks with an additional sampling day on week 6 (16 total sampling dates) that spanned the months of October and November 2023. Three individual samples of both the BJ contact surface (*N* = 96) and meat (*N* = 96) were collected from the belly processing line during normal production at the beginning and end of the first shift on each sampling date. The same sampling protocol was followed for the BBT contact surface (*N* = 96) and meat (*N* = 96) samples collected from the Boston butt shoulder processing line. Sampling at the beginning of the shift occurred at approximately 6:00 a.m. and at the end of the shift at 1:30 p.m. Due to the location and distance between the processing lines, sampling of both the meat and contact surfaces occurred on the BBT line first, followed by sampling of the meat and contact surfaces of the BJ line.

Meat samples for both the BJ and BBT consisted of approximately 1,600–2,000 g of trim meat that was aseptically removed from either the conveyor (BJ) or stainless steel chute (BBT). The three samples collected at each shift time point were collected in succession and immediately placed into individual sterile 19 cm × 38 cm Whirl-Pak bags (Whirl-Pak, cat. no. B01451). Each sample was placed in a cooler with ice packs and immediately transferred to the Hormel Foods Research and Development Laboratory in Austin, MN, for refrigerated storage (4.5°C) overnight. Total transit time between the plant and the laboratory was approximately 15 minutes. On the following day, a sterile 20 cm × 61 cm MicroTally spun polymer cloth mitt (Fremonta, San Jose, CA, cat. no. MT-100) was aseptically placed in the bottom of an oversized sterile 30 cm × 61 cm poly bag (Veritiv, cat. no. 000885), and the entire contents of the meat sample (approximately 1,600–2,000 g) were aseptically transferred to the bag that contained the mitt. The oversized bag, meat, and MicroTally mitt were sealed without vacuum using a Multivac C400 packaging machine (Multivac SEPP Haggenmuller SE & C. KG, Wolfertschwenden, Germany). Once sealed, the meat and MicroTally mitt were vigorously hand mixed for 1 minute to adequately expose the mitt to meat purge and all meat surfaces in the sample. The MicroTally mitt was then aseptically removed from the bag and placed in an 18 cm × 28 cm Whirl-Pak bag. MP Media (BAX Systems, cat. no. MED2003) was prewarmed to 42°C, and 200 mL was aseptically added to the MircoTally mitt and stomached at high speed for 30 seconds (BioMerieux AES, SMASHER, cat. no. AESAP1064).

Contact surface swab samples were collected using an EZ Reach polyurethane sponge split swab with 20 mL HiCap Neutralizing Broth (World Bioproducts, cat. no. EZ-SP-20HC-PUR) from either the transfer conveyor (BJ) or stainless steel chute (BBT). Each contact surface location was swabbed over a 10 cm × 10 cm area using a vertical, diagonal, and then vertical swabbing pattern across the surface. Immediately after sampling, the split swab was broken, and each swab was placed into individual Whirl-Pak bags for further analysis. Since the BJ line conveyor was moving during sampling, the same area of the conveyor was not sampled twice at each time point. However, the same three distinct but adjacent locations of the stainless steel chute were sampled for the BBT at each time point. After sampling, each swab was placed in a cooler with ice packs and immediately transferred to the Hormel Foods Research and Development Laboratory in Austin, MN, for refrigerated storage (4.5°C) overnight. After overnight storage, 99 mL of sterile Butterfield’s phosphate buffer (World BioProducts, cat. no. NLD-99BFD) was added to the swab and Whirl-Pak bag used for sample collection. The swab was repeatedly squeezed for 10 seconds to release the contents of the swab into the buffer.

### Sample preparation, DNA extraction, library preparation, and sequencing

Aliquots from the MicroTally mitt meat homogenate and contact surface swab suspensions were used for DNA extraction. Immediately after the meat samples were stomached, a 10 mL aliquot of the homogenate was portioned into a 50 mL conical tube. For the contact surface swabs, a 38 mL aliquot was taken from the swab suspension and portioned into a 50 mL conical tube. Both samples were immediately centrifuged at 13,000 × *g* for 12 minutes at 4°C. The supernatant was removed, and the remaining pellet was retained and stored at −80°C until DNA extraction. Approximately 20 minutes elapsed between the addition of the media to both sample types and storing of both the meat and contact surface sample pellets at −80°C.

Both meat and contact surface samples were subjected to DNA extraction using the DNeasy PowerFood Microbial Kit (Qiagen, cat. no. 21000-100-MON, 2.0 mL bead tube, Hilden, Germany) and automated on the QiaCube Connect instrument (Qiagen, cat. no. 9002864, Hilden Germany). Pelleted samples were selected and thawed for 20 minutes in a biosafety cabinet sterilized with UV light and 70% ethyl alcohol. Once thawed, 450 µL of prewarmed (55°C) MBL reagent was added to each rinsate pellet and vortexed at high speed until the pellet was homogeneously suspended. The remaining DNA extraction procedures continued according to the manufacturer’s instructions. The QiaCube instrument was run 33 times, with a maximum sample throughput of 12 samples per run. One extraction blank was included at the beginning of each extraction day (10 days total) and consisted of 450 µL of prewarmed MBL reagent that was then processed alongside meat samples through the rest of the PowerFood extraction procedure. Two positive controls that contained a known composition of eight bacterial and two fungal species (Zymo Research Corp., D6310) were included as an internal control at the end of the last extraction run. All DNA extractions were randomly ordered, and 20 µL of DNA from each sample was transferred onto PCR plates (four total). Plates were covered with an adhesive seal and submitted to the University of Minnesota Genomics Core for library prep and sequencing.

The copy number of the 16S rRNA gene amplicon per microliter in each sample was determined using the KAPA HiFi Hot Start polymerase (Roche, Indianapolis, IN) and QuantStudio 5 real-time PCR machine (Applied Biosystems, Waltham, MA) at 30 PCR cycles to ensure adequate copies of the 16S rRNA gene/μL for sequencing (42). The limit of detection for the qPCR assay was 10 16S rRNA gene amplicons/μL (42). Libraries were prepared by amplifying the V3-V4 region of the 16S rRNA gene, using primers V3_357F_Nextera: CCTACGGGAGGCAGCAG and V4_806R_Nextera: GGACTACHVGGGTWTCTAAT (42). The same primers and reagents used for qPCR were also used to make the amplicons for 16S sequencing. Prepared libraries were sequenced to an expected depth of 150,000 paired-end reads per sample on an Illumina NextSeq 2000 instrument (Illumina Inc., San Diego, CA) using a P1 flow cell (2 × 300 base pair) reagent kit (Illumina Inc., San Diego, CA).

### Bioinformatics

Bioinformatic analyses were conducted using R Statistical Software (v.4.3.2; R Core Team 2021), and plots were generated using the ggplot2 package. Adapter sequences and primers were first removed from the raw sequences using Cutadapt v.4.0, with zero-length reads removed by filtering to minimum length = 1 (43). Raw sequences were then processed through the DADA2 (v.1.30.0) pipeline for quality filtering, denoising, and microbial inference (44). Forward and reverse reads were truncated to lengths of 250 and 230 base pairs, respectively, based on the distribution of quality scores. Forward and reverse reads with expected error rates exceeding 3 and 4 base pairs, respectively, were discarded. Forward and reverse reads were merged, and chimeras were identified and removed using the removeBimeraDenova function. The merged, chimera-free ASVs were aligned to the SILVA reference database v.138.1 (45) for taxonomic assignment. Species identification was performed using the addSpecies function in DADA2*,* with species aligned and assigned using the SILVA Species Assignment v.138.1.1 database (45). The ASV count matrix, taxonomy table, and sample metadata were combined to create a phyloseq object for further microbiota analysis using the phyloseq (v.1.46.0) R package (46).

### Identification and removal of contaminants

Identification and removal of potential sequence contaminants was conducted using the decontam v.1.22.0 (47) package in R. The phyloseq object was subjected to three methods of contaminant identification: Frequency, Prevalence, and Combined. Thresholds of 0.1, 0.5, and 0.39 were used in the isContaminant function for the Frequency, Prevalence, and Combined methods, respectively. The distribution of identified contaminant ASVs was used to select the most appropriate contaminant identification method. ASVs identified as contaminants were removed from the final phyloseq object. Visualization of contaminants for the Frequency, Prevalence, and Combined methods was conducted utilizing the methods and base R code provided by Dean and Deng et al. (48).

Reads from the positive controls were aligned to the manufacturer’s reference database (https://s3.amazonnews.com/zymo-files/BioPool/ZymoBIOMICS.STD.refseq.v2.zip) and processed through Cutadapt and DADA2 as described above, but were not processed through decontam to remove potential contaminants. The theoretical composition of the mock samples (provided by the manufacturer) was compared to the observed composition of the positive controls.

### Microbiota analysis

The contaminant-free phyloseq object was subsetted to only include ASVs that were identified at the Domain level, thus removing all ASVs identified as NA at the Domain level. Five samples (three meat, two contact surface) were determined to be outliers and removed from further analysis, due to either low 16S rRNA gene amplicon copy number or low overall sequencing reads.

To assess beta diversity, ASV counts were first normalized using cumulative sum scaling (CSS) from the cumNorm function in the metagenomeSeq v.1.43.0 package. Bray-Curtis dissimilarities were calculated using the distance function in the phyloseq package. NMDS was used to ordinate the Bray-Curtis dissimilarities using the ordinate function in the phyloseq package. The effects of processing line, sample type, and production schedule were tested using PERMANOVA as implemented in the adonis2 function in the vegan v.2.6.4 package, with the default number of permutations (i.e., 999). NMDS and spider plots were generated using the ggplot2 package. Both individual and interaction effects for processing line and sample type were included in the PERMANOVA model. To assess the effect of the production schedule, samples were first subsetted by processing line and sample type, and then shift time and production date were included in the PERMANOVA model. Associated *R*^2^ values for key variables were visualized via a chart using the gt package.

Abundance was calculated as the number of sequencing reads aligned to each ASV within each sample. ASVs were grouped at the genus level using the tax_glom function in the phylsoeq package. For relative abundance, ASV counts were first transformed using the transform_sample_counts function in the phyloseq package and then grouped at the genus level.

Commonality of taxa across various sample types was visualized using the UpSetR and MicrobiotaProcess packages (49, 50). The phyloseq object was first subsetted to include only ASVs with taxonomic identification at the genus level, and then was conducted both at the individual ASV and genus levels.

Differential abundance of key genera associated with food safety or spoilage was calculated using the MaAsLin2 v.1.16.0 package (51) using the default “LM” analysis method and specifying a multivariate model with processing line, sample type, and shift time as fixed effect variables. Briefly, ASV counts were first normalized using CSS from the cumNorm function in the metagenomeSeq v.1.43.0 package and grouped at the “genus” level using the tax_glom function in the phyloseq package. Output from MaAsLin2 was transformed to a log scale and statistical significance determined at qval ≤ 0.05. For visualization purposes, a selection of key genera known to be associated with food safety or spoilage was reported, with the full results provided in the Supplementary data.

The SourceTracker v.1.0.1 package was used to identify potential sources of bacteria detected within the meat and contact surface samples by processing line and shift time (52). The default rarefaction = 1,000 setting was used for each of the analyses.

## Data Availability

All data sets are available at the NCBI SRA database under BioProject accession number PRJNA1178689.

## SUPPLEMENTAL MATERIAL

Data Set S1 aem.00044-25-s0001.csv MaAsLin2 differential abundance of meat samples.

Data Set S2 aem.00044-25-s0002.csv MaAsLin2 differential abundance of contact surface samples.

Data Set S3 aem.00044-25-s0003.csv MaAsLin2 differential abundance of BJ meat and contact surface samples.

Data Set S4 aem.00044-25-s0004.csv MaAsLin2 differential abundance of BBT meat and contact surface samples.

Supplemental material aem.00044-25-s0005.pdf Figures S1 to S6; Table S1.

ASM does not own the copyrights to Supplemental Material that may be linked to, or accessed through, an article. The authors have granted ASM a non-exclusive, world-wide license to publish the Supplemental Material files. Please contact the corresponding author directly for reuse.
