# Supplementary material for: Fresh pork microbiota is temporally dynamic and compositionally diverse across meat, contact surfaces, and processing lines in a pork processing facility
Source: Appl Environ Microbiol. 2025 Apr 3;91(4):e00044-25. doi: 10.1128/aem.00044-25 (PMC12016530; doi:10.1128/aem.00044-25)
Supplement: Supplemental material — Figures S1 to S6; Table S1. [file aem.00044-25-s0005.pdf]

**Figure S1** 16S rRNA gene amplicon sequencing depth by sample type, before and after QC filtering and decontamination using *decontam*

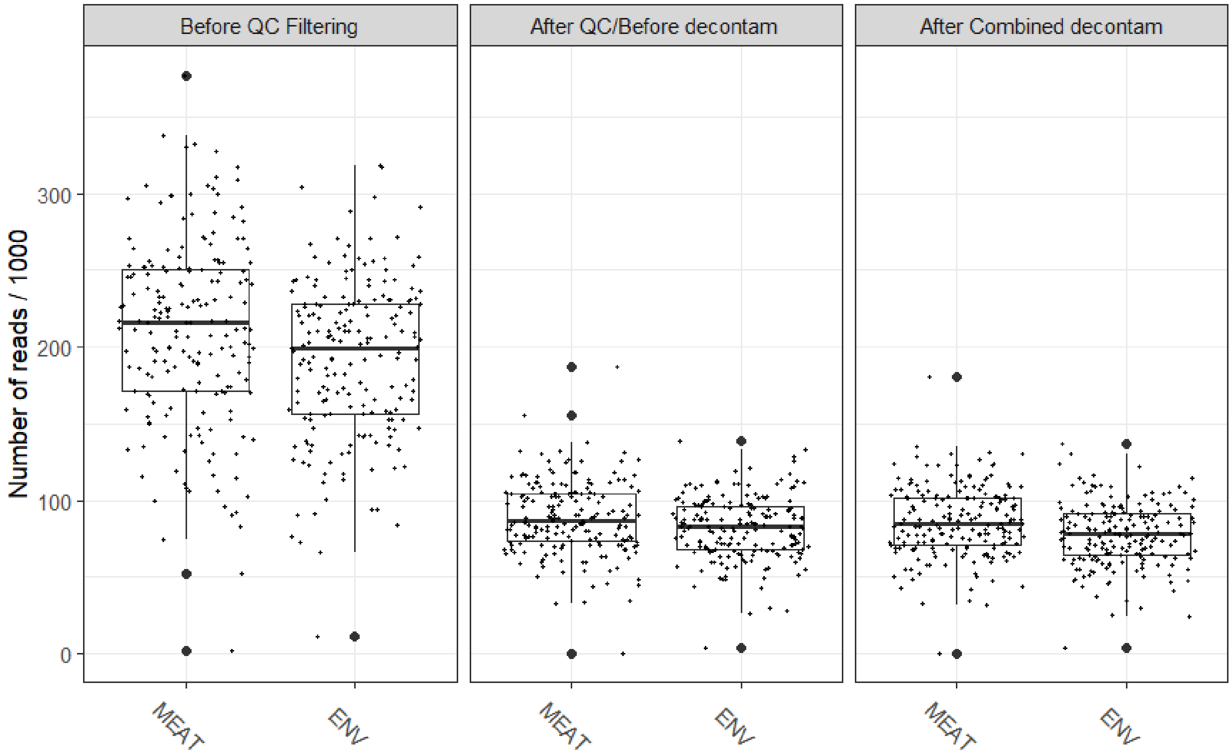

**Figure S2** Genus-level relative abundance for the positive control sample processed in this study (“S282”, “S26”) and the theoretical composition of the Zymo Mock Control (“Mock”)

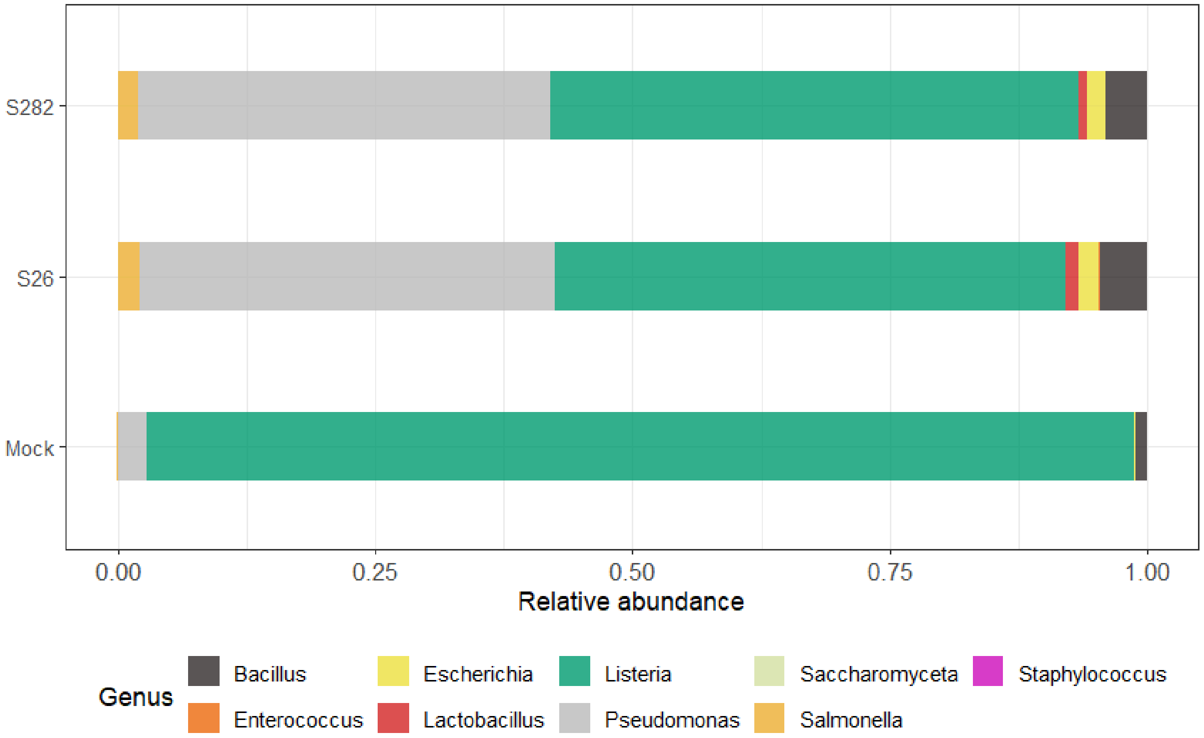

**Figure S3** Sequence reads abundance of ASVs removed as contaminants from all sample types using the Combined method in the *decontam* package. ASVs removed were combined at the genus level using the *aggregate_rare* function in the *microbiome* package.

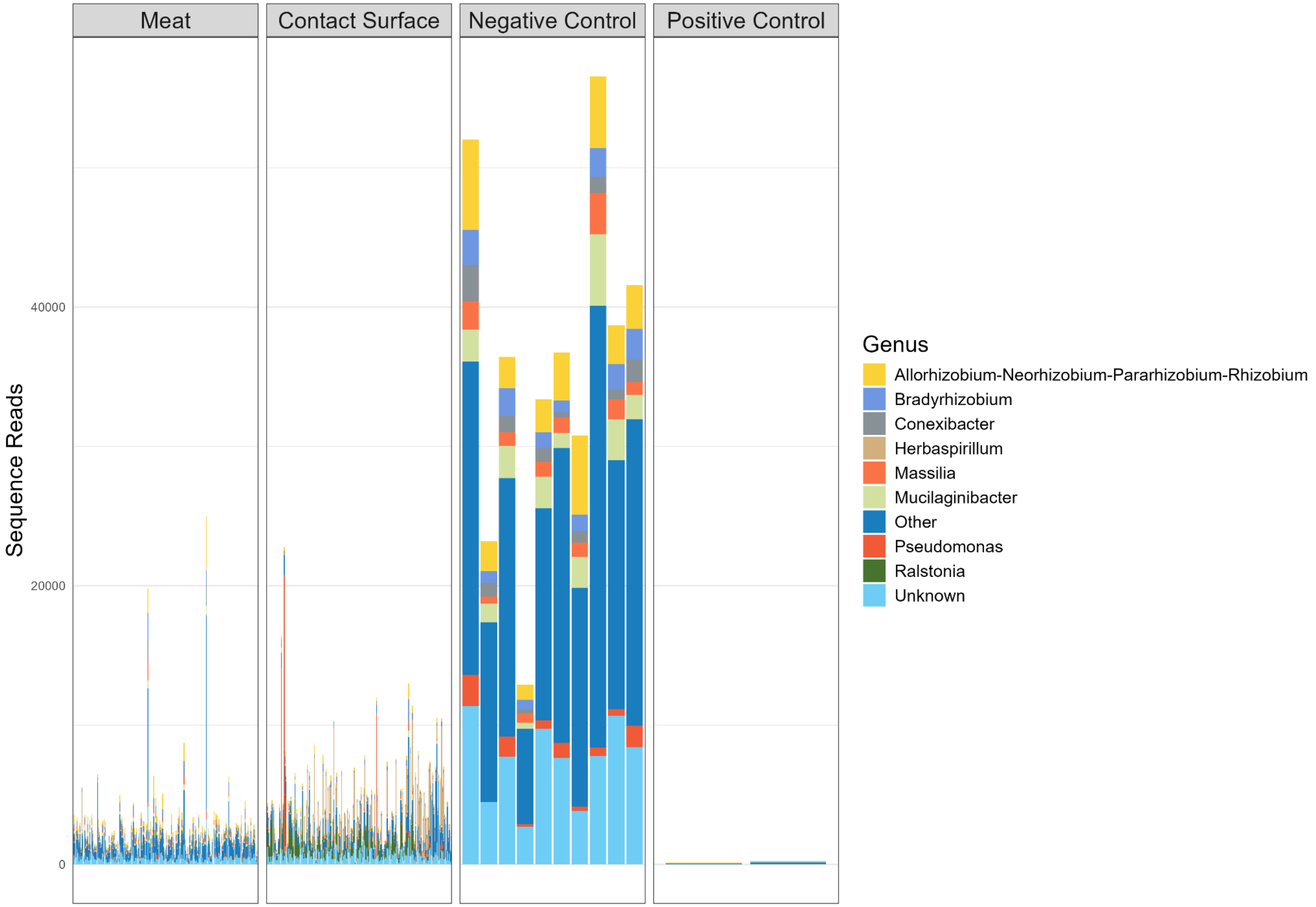

**Figure S4** Compositional description of the microbiota (ASV-level) by processing line and sample type. (A) Non-metric multidimensional scaling ordination plot of Bray Curtis dissimilarities for both meat and contact surface samples for Bootjack Trim (yellow) and Boston Butt Trim (blue) processing lines. (B) Non-metric multidimensional scaling ordination plot of Bray Curtis dissimilarities of both the BJ and BBT processing lines for contact surface (gray) and meat (red) samples. (C) Non-metric multidimensional scaling ordination plot of Bray Curtis dissimilarities for the BJ contact surface (purple), BJ meat (red), BBT contact surface (green), and BBT meat (orange).

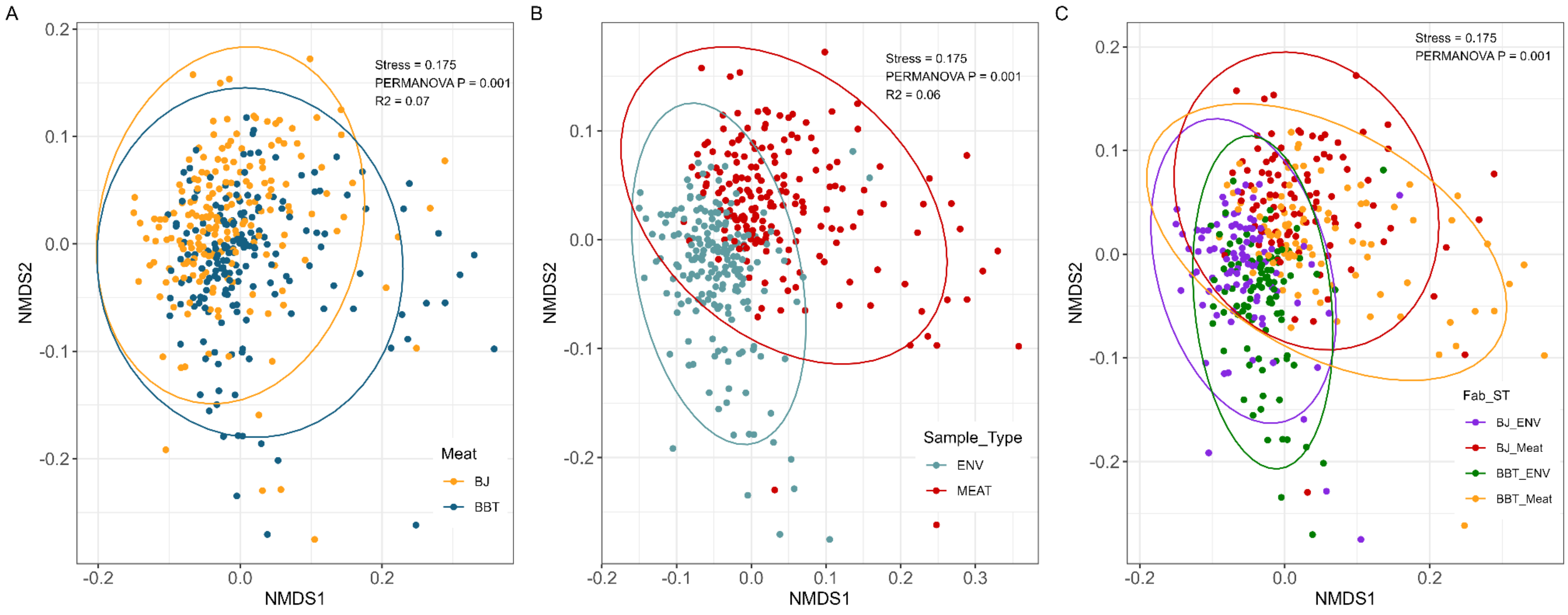

**Figure S5** UpSetR plots showing the intersection of common amplicon sequence variants (ASVs) for the Bootjack Trim (A) and Boston Butt Trim (B) across meat or contact surface (ENV) samples at either the beginning (Beg) or end (End) of the production shift. The total number of intersecting ASVs is represented on the y-axis. Each individual combination of sample type and shift time is denoted on the x-axis, with dots representing the combination of each factor included in the total number of intersecting ASVs (A) or genera (B) reflected in each bar. The total number of ASVs for each level of sample type and shift time is reflected in the Set Size bar chart.

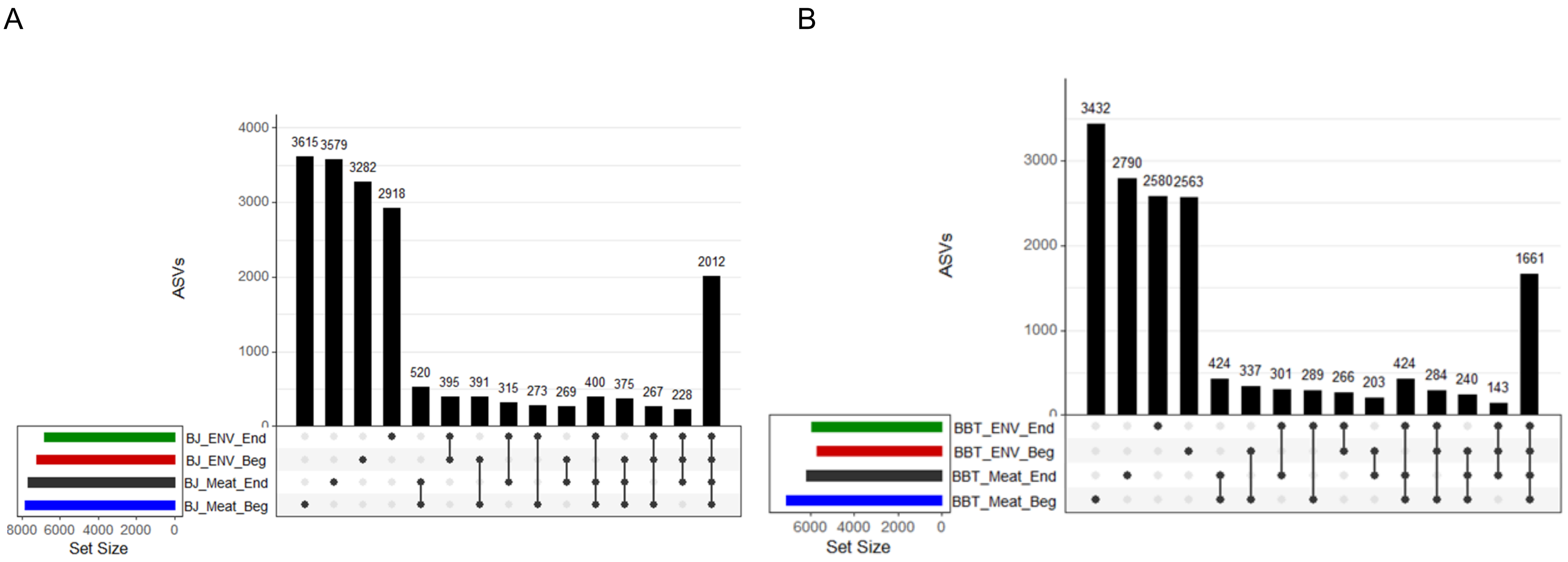

**Figure S6** Non-metric multidimensional scaling (NMDS) ordination of Bray Curtis dissimilarities by processing line and processing date. (A) Bootjack Trim meat (B) Bootjack Trim contact surface (C) Boston Butt Trim meat (D) Boston Butt Trim contact surface

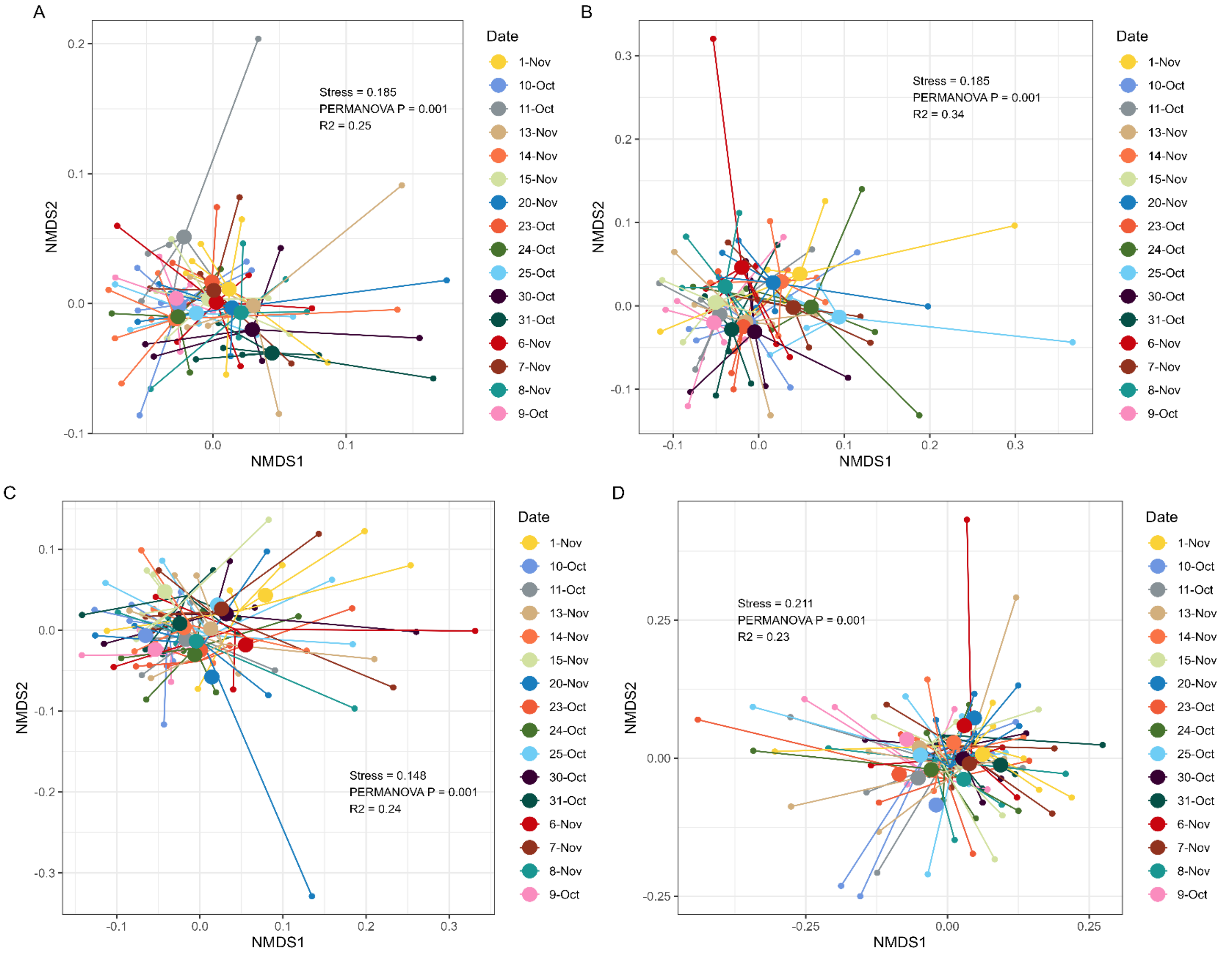

**Table S1** Taxa Identified at Each Taxonomic Level By Sequencing Reads and Identified ASV's.

| Taxonomic_Level | Reads.ID | Percent.Reads.ID | Taxa.ID.ASV. | Percent.Taxa.ID.ASV. |
|---|---|---|---|---|
| After_ Decontam | 31,388,106 | 100.0% | 127,440 | 100.0% |
| Domain | 29,459,844 | 93.9% | 118,092 | 92.7% |
| Phylum | 23,018,822 | 73.3% | 59,517 | 46.7% |
| Order | 21,971,891 | 70.0% | 50,370 | 39.5% |
| Genus | 19,466,314 | 62.0% | 29,622 | 23.2% |
| Species | 3,065,595 | 9.8% | 1,667 | 1.3% |
